# Accuracy of POC testing systems for HPV screening: the importance of disease prevalence and characteristics of the screened population

**DOI:** 10.1186/s13027-024-00629-9

**Published:** 2024-12-21

**Authors:** Paolo Giorgi Rossi, Guglielmo Ronco

**Affiliations:** 1 Azienda USL – IRCCS di Reggio Emilia, Via Amendola 2, 42122 Reggio Emilia, Italy; 2 Centre for Cancer Epidemiology and Prevention (CPO), Turin, Italy

**Keywords:** Cervical cancer, Cervical intraepithelial neoplasia, Screening, Human papillomavirus, Accuracy

## Abstract

Infectious Agents and Cancer journal has recently launched a new collection of papers about “Point-of-Care (POC) for HPV-related genital cancers” putting together some interesting works on the accuracy of HPV tests for screening. This editorial initiative gave us the opportunity to reflect on the relations between accuracy measures, prevalence and characteristics of the tested population in the case of HPV-based screening. In screening test evaluation, we look at the clinical accuracy of the test as an intrinsic characteristic of the assay, which interacts with the characteristics of the population, the result being the screening performance. In the case of HPV testing, the clinical accuracy should be conceptualized in two steps, the analytical accuracy of the assay for HPV infection and the biological link between HPV infection and the target disease, i.e. the high-grade cervical intraepithelial neoplasia (hgCIN). This approach highlights that just a few false positive cases result from a lack of analytical specificity while most derive from women who have the infection but it did not progress to hgCIN. In addition, increasing prevalence of hgCIN results in relevant increases of PPV only if due or associated with exposures which increase the progression from infection to hgCIN or the duration of the latter; while an increase due to a higher prevalence of HPV infection would only marginally affect PPV. This approach may help in modelling the performance of HPV-based cervical screening.

Infectious Agents and Cancer journal has recently launched a new collection of papers about “Point-of-Care (POC) for HPV-related genital cancers” putting together some interesting works on the accuracy of HPV tests for screening. This editorial initiative gave us the opportunity to reflect on the relations between accuracy measures, prevalence and characteristics of the tested population in the case of HPV-based screening.

In screening test evaluation, we look at the clinical accuracy of the test as an intrinsic characteristic of the assay, which interacts with the characteristics of the population, the result being the screening performance. In the case of HPV testing, the clinical accuracy should be conceptualized in two steps, the analytical accuracy of the assay for HPV infection and the biological link between HPV infection and the target disease, i.e. the high-grade cervical intraepithelial neoplasia (hgCIN). This approach highlights that just a few false positive cases result from a lack of analytical specificity while most derive from women who have the infection but it did not progress to hgCIN. In addition, increasing prevalence of hgCIN results in relevant increases of PPV only if due or associated with exposures which increase the progression from infection to hgCIN or the duration of the latter; while a simple increase due to a higher prevalence of HPV infection would only marginally affect PPV. This approach may help in modelling the performance of HPV-based cervical screening.

Since 2013, when WHO guidelines recommended HPV screening also for low-income countries [1], many pilot studies have been conducted to find a feasible and sustainable screening strategy in sub-Saharan Africa [2–5]. Nevertheless, HPV-based screening is far from being implemented on a large scale in the populations needing it most. One of the main barriers is the cost of the test. Furthermore, developing a “point of care” assays that can be used for test-and-treating or test-triage-and-treating strategies is necessary. A prerequisite for implementing these strategies is the availability of HPV and triage assays that can give the results in a few hours.

Three studies recently published in the Infectious Agents and Cancer journal deal with the accuracy of HPV testing for cervical screening. Two compared a new HPV assay with the Xpert HPV assay [3, 6]. Dreyer and colleagues tested different options for primary screening including the Cobas HPV assay [7], a longitudinally validated test [8]. All have been conducted in sub-Saharan Africa and thus a large proportion of included women was immunosuppressed because of HIV infection. We start with the results of such studies to share some considerations about the implications that the conceptual framework of the causal links between test target, disease and characteristics of the population, may have on the design and interpretation of studies on HPV test accuracy.

It is worth noting that both the specificity and positive predictive value (PPV) of HPV testing for hgCIN change when moving from HIV-negative women (HNW) to HIV-positive women (HPW). In a sample of HPW living in Botswana with a 16% prevalence of CIN grade 2 or more severe (CIN2 +) the specificity of the reference and the comparator tests were 45% and 51% respectively while PPVs were 29% and 27% respectively [6]. Similarly, the second study included only HPW from Malawi, with a 16% CIN2 + prevalence. The observed specificity values for the reference and comparator tests were 28% and 34%; the PPVs were 19% and 21% respectively [3]. Dreyer and colleagues conducted their study in a population with a very high prevalence of cancer and an unexpectedly high prevalence of CIN2 + negative to any screening test, including HPV. Among HNW, the PPV of HPV testing for CIN2 + was 30% and its specificity 82% while among HPW PPV was 39% and specificity 62%. The prevalences of CIN2 + were 10% and 23% respectively and those of HPV positivity were 23.5% and 48.5% respectively [7]. It is also worth reminding that, based on a systematic worldwide review of HPV clinical accuracy published in 2008 [9] we found that the variation in specificity of HPV testing for CIN2 + was almost entirely explained by a linear negative function of HPV prevalence in the study population (R^2^ = 0.98) while PPV was weakly related to HPV prevalence (R^2^ = 0.38) with both presenting high values in low-income countries [10].

Understanding such results needs conceptually splitting the two-by-two accuracy table of test results and disease status in a first one considering the analytical accuracy of the HPV test for its target (i.e. the presence of HPV infection) and a second one, considering the association between the test target (HPV infection) and the disease (hgCIN). In the analytical accuracy table, it is reasonable to assume that the sensitivity and specificity of the assay are stable across populations. If this is the case, then the test’s PPV for HPV infection will increase with increasing viral prevalence in the population. In the second table, given the causal link between HPV and hgCIN, the probability of having a hgCIN when the virus is present and the same probability when the infection is not present are also expected to be quite stable. As HPV is a virtually necessary cause of cervical precancer and cancer, then the latter probability is virtually null (i.e. NPV = 1). The probability of progression from infection to hgCIN is typical of each genotype with strong differences among them [11]. However, other factors can affect such progression probability (mainly immunosuppression) or duration of disease, i.e. how long the hgCIN lasts, (which may be influenced mainly by age and by the probability of having been screened and treated before) and thus, as a result, the PPV of the HPV infection for hgCIN, i.e. the biological link, canges accordingly.

With a probability of HPV-infected women carrying hgCIN around 50% and an analytical specificity of 98% (Fig. 1) false positives due to analytical failure (i.e. women who are positive for the test but do not carry the infection) are relatively few while most of the false-positive results occur in women who carry the infection but not hgCIN. An increase in HPV prevalence without changing the genotype mix nor the prevalence of progression modifiers will not change the PPV despite the increased absolute hgCIN prevalence.

**Fig. 1:**
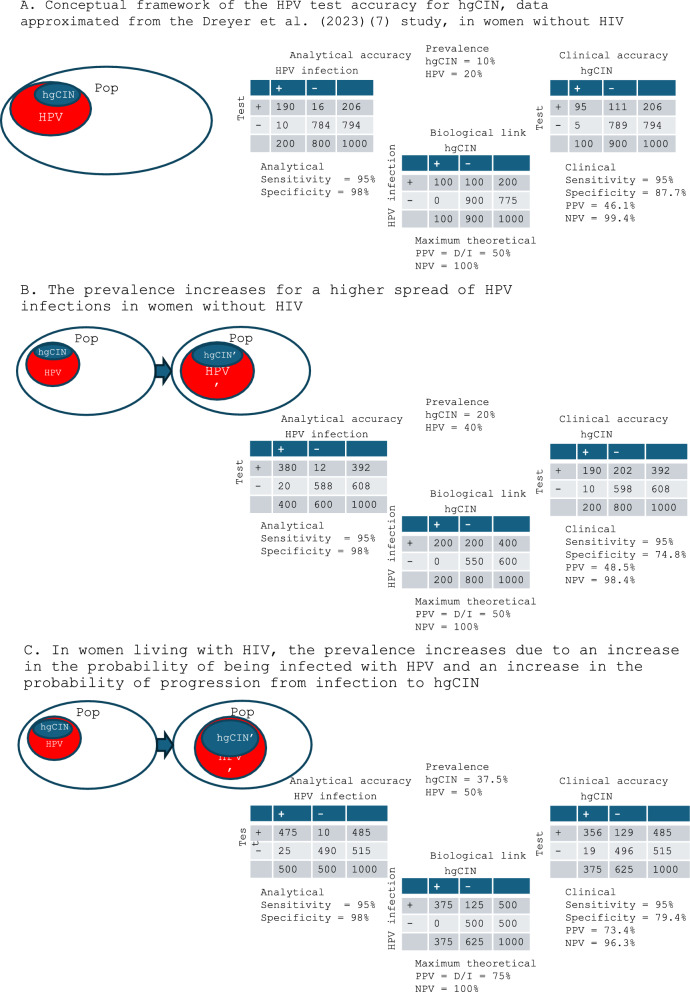
Conceptual framework of HPV testing accuracy for CIN2 +. Panel **A** reports the base case. The up-left table represents the analytical accuracy, i.e. the ability of the test to correctly identify the virus, In the own-central table we represent the association between HPV infection and CIN2 + in women without HIV using data approximated from the Dreyer et al. 2023 study (7). We considered HPV a necessary cause. The up-right table represents the clinical accuracy of the HPV test for CIN2 + as predicted by the conceptual model. Panel **B** reports a theoretical population with an increased prevalence of CIN2 + due only to an increase in the prevalence of HPV infection, i.e. the biological link does not change. In this case, clinical positive predictive value (PPV) only slightly increases, while clinical specificity decreases. Panel **C** reports a population in which the increase in prevalence is due both to an increase in infections and to an increase in the probability of progression from infection to CIN2 +, i.e. the biological link changes. This scenario may be representative of the women living with HIV. In this scenario, clinical PPV increases and clinical specificity decreases

In populations where genotype mix, frequency of modifiers of progression to CIN2 + and screening histories are stable, increases of CIN2 + prevalence due only to increase of HPV prevalence are expected to result in minimal, if any, increase in PPV for CIN2 + . However, as all women who acquire HPV infection exit from negatives but only part will develop CIN2 + then specificity will necessarily decrease (Fig. 1 panel B).

In the case of HPW, the probability of acquiring HPV infection is higher and the probability of having a CIN2 + given the infection, i.e. the biological link, is also higher than in women without HIV [12, 13], thus the clinical PPV is slightly higher, but the largest change is in specificity, as a consequence of the change in HPV infection prevalence (Fig. 1 panel C). These considerations have several implications for interpreting the performance indicators in screening programs and for actions to be put in place to improve them. They also have implications on how to model the impact of different screening strategies in different populations.

This example underlines that authors were very correct in considering separately HPW and HNW but also shows that accurately predicting how the clinical accuracy of an HPV assay will change in different populations is not always trivial. For example, many professionals plausibly expect that PPV will increase at any increase in HPV prevalence, while we just showed that this will happen in some cases but not in others. This is particularly important for validating and assessing utility of PoC HPV tests, because the balance between desirable and undesirable effects of the test-and-treat or test-triage-and-treat strategies that could be implemented depends on the actual PPV in the screened population.

## Data Availability

No datasets were generated or analysed during the current study.

## Abbreviations

hgCIN: High-grade Cervical Intraepithelial Neoplasia
CIN2 +: Cervical Intraepithelial Neoplasia grade 2 or more severe
HNW: Human Immunodeficiency Virus negative woman
HPV: Human Papillomavirus
HPW: Human Immunodeficiency Virus positive woman
NPV: Negative Predictive Value
POC: Point of Care
PPV: Positive Predictive Value
